# Supplementary figures and images for: Telomerase RNA plays a major role in the completion of the life cycle in Ustilago maydis and shares conserved domains with other Ustilaginales
Source: PLoS One. 2023 Mar 23;18(3):e0281251. doi: 10.1371/journal.pone.0281251 (PMC10035886; doi:10.1371/journal.pone.0281251)

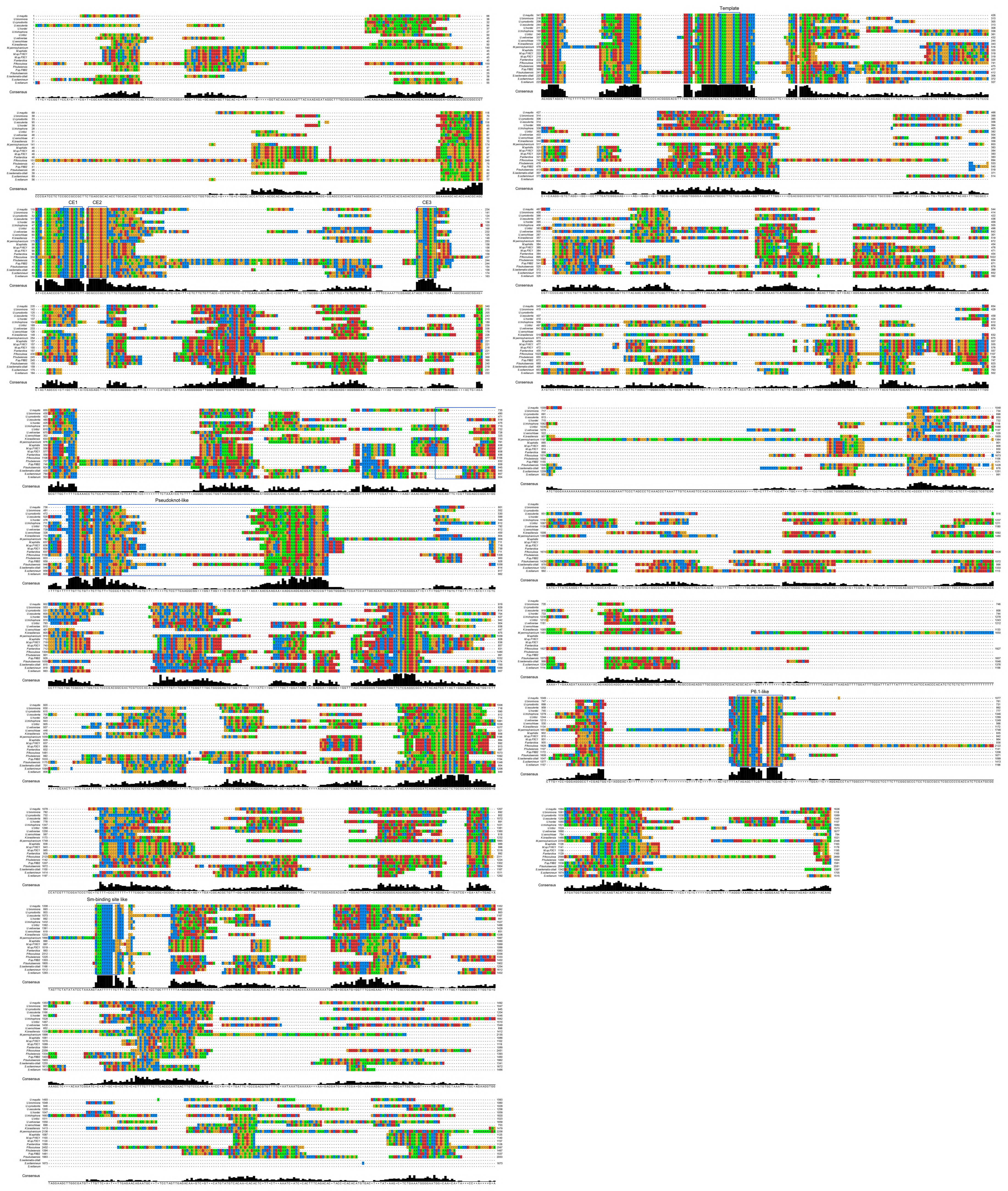

Supplement: S1 Fig — A. Multiple alignment of intergenic sequences where the CE1, CE2, and CE3 domains of the gene encoding the TER subunit are located in boxes. B. Multiple alignment of intergenic sequences of the gene encoding the TER subunit in the section boxing the putative template domain. C. The multiple alignment of intergenic sequences shows the pseudoknot-like domain located in a box on the putative genes encoding the TER subunit. D. Multiple alignment of intergenic sequences showing the P6.1-like domain of the gene encoding the TER subunit in a box. E. Sm-binding site-like domain boxed on the multiple alignment of intergenic sequences of the gene encoding the TER subunit. F. Final segments of intergenic sequences of the multiple alignment of the putative genes encoding the TER subunit in several Ustilaginales. (TIF) [file pone.0281251.s001.tif]

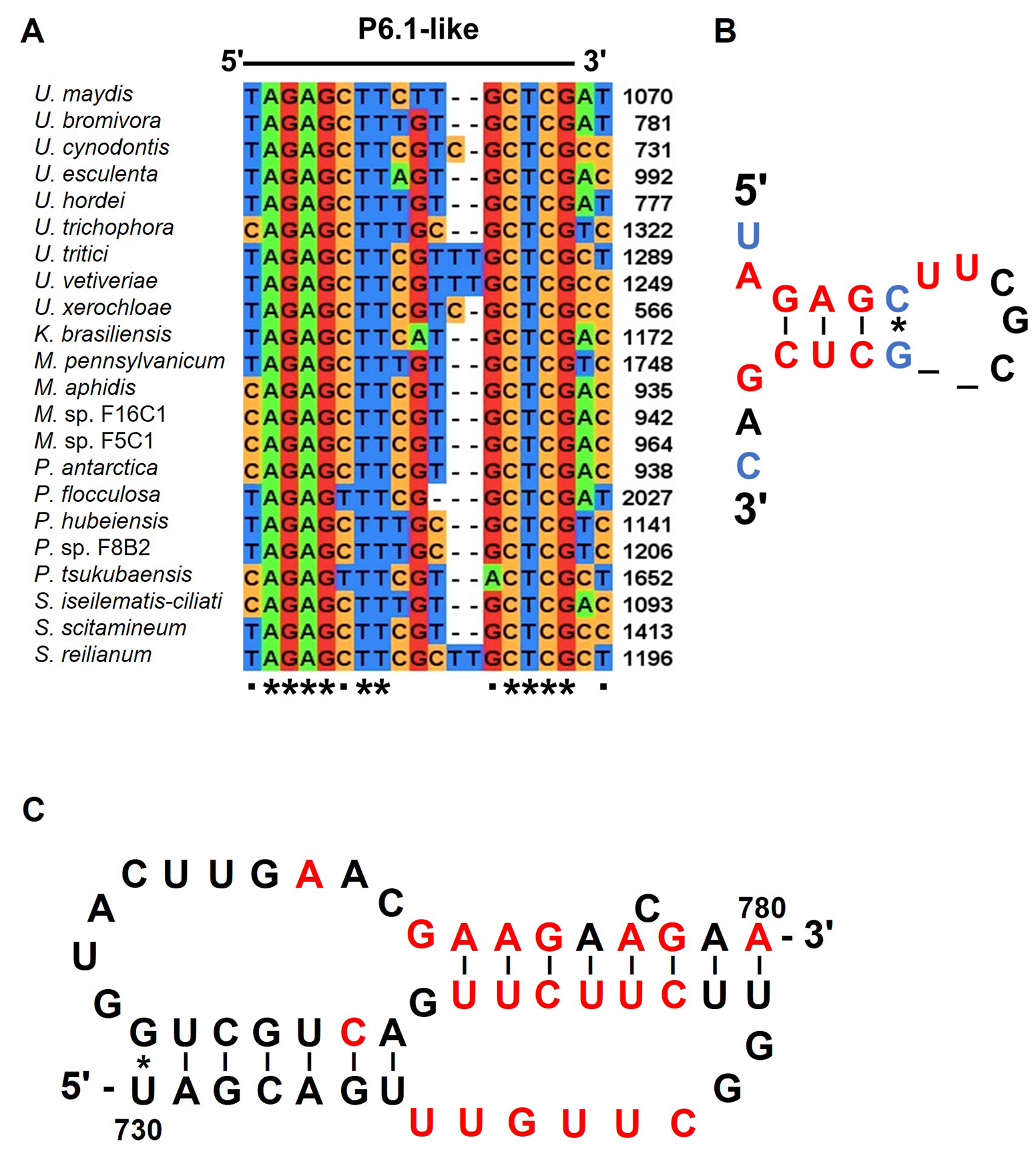

Supplement: S2 Fig — (A) MSA of the conserved region reminiscent of the P6.1 sequence in vertebrates. (B) Secondary consensus structure of the P6.1 hairpin according to the RNAalifold program. Nucleotides conserved in all species are in red, and nucleotide transitions among species are in blue; base pairings supported by covariations are marked by asterisks. (C) Structure of the pseudoknot domain. Potential folding of the conserved region resembling CS3 and CS4. Red nucleotides represent the conserved residues in at least 80% of the species. (TIF) [file pone.0281251.s002.tif]

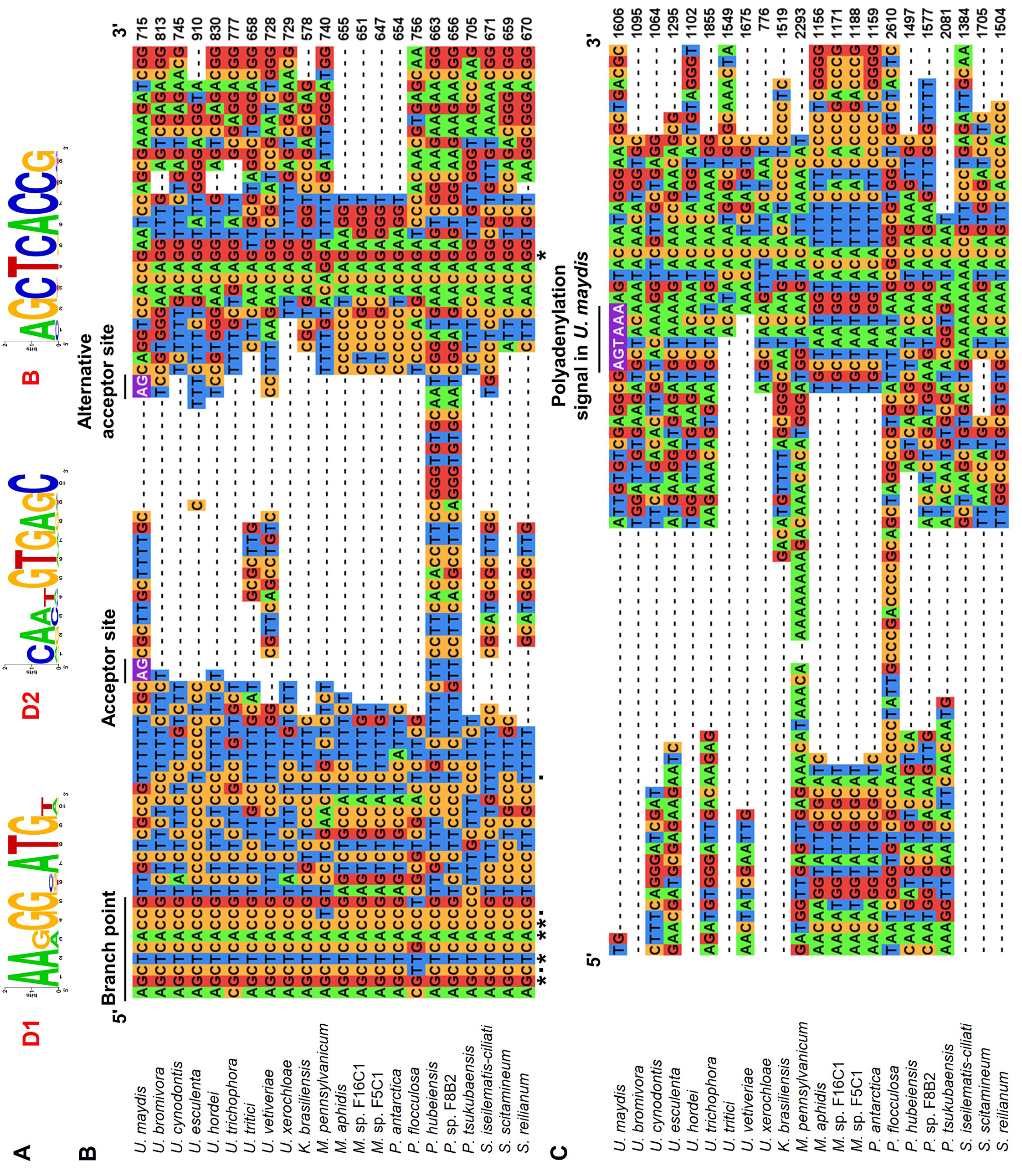

Supplement: S3 Fig — (A) WebLogo of the splicing sites of UMAG_03168. D1 corresponds to the sequence of the donor points used in the processing of KIS68596 and ter1-i4, D2 corresponds to the donor site used for the processing of isoforms 2 and 3 of ter1, and B corresponds to the branch site sequence used in the processing of the different isoforms observed. (B) Multiple alignment of the branch point location and acceptor sites used in U. maydis for the processing of transcripts. The pairs of AG bases used as acceptor sites are highlighted in purple, showing that the alternative acceptor site used in the processing of ter1-i3 is located near a region with AG residues conserved across Ustilaginales species. (C) Multiple alignment of the 3’ end of the ter1 locus. Final region of the ter1 locus where a possible polyadenylation signal was identified, which is located in a region that is rich in A residues in Ustilaginales species. (TIF) [file pone.0281251.s003.tif]

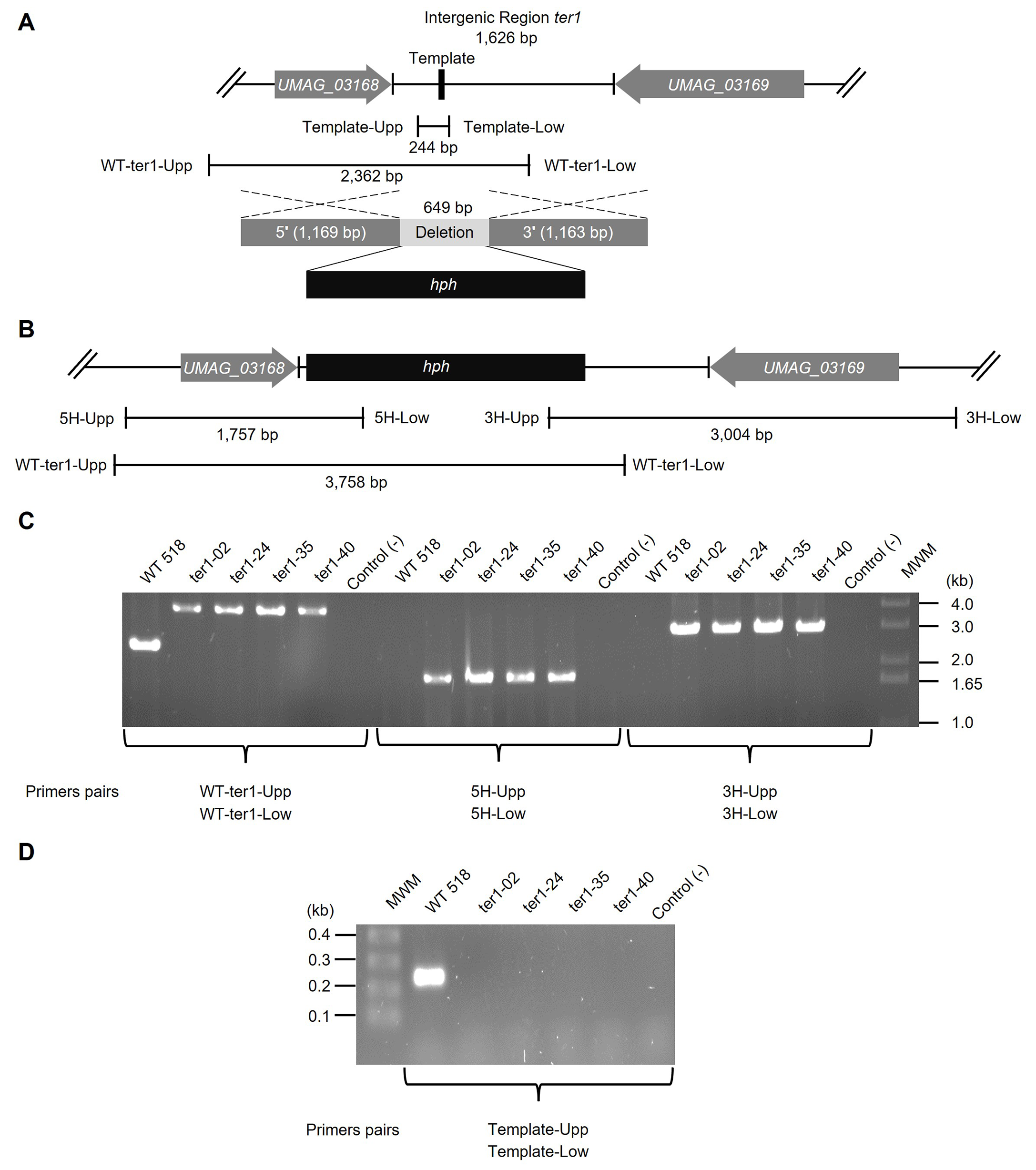

Supplement: S4 Fig — Target sequence deletion was tested by PCR assays in which different pairs of primers were designed for the WT strain and interrupted transformers. (A) locus interruption strategy and alignment diagram of the primers designed to check the integrity of the locus in the WT strain. (B) Diagram of the alignment of the primers to verify the replacement of the target sequence with the hph gene sequence. (C) The replacement of the locus was analyzed in 4 transformants: ter1-02, ter1-24, and ter1-35 and ter1-40. They show an increase in the size of the amplified size corresponding to the size of the replacement of the target sequence by the gene hph. The WT strain produces the expected amplicon of 2,362 bp, whereas the negative control does not produce any amplification. The integration of the selection marker sequence was checked at both ends of the recombination site using pairs of primers aligning within the hph gene sequence and outside the recombination site, and amplification products of the expected size were obtained in the ter1 mutants. In the integration check from the 5’ end, the expected amplification product of 1,757 bp was obtained from mutants ter1-02, ter1-24, ter1-35, and ter1-40. In the integration check from the 3’ end, the expected amplified product of 3,004 bp was obtained from mutants ter1-02, ter1-24, ter1-35, and ter1-40. Amplification was not obtained from the WT strain or negative controls. (D) Corroboration of the absence of the target sequence. Pairs of primers aligning within the deleted region were used. A fragment of 244 bp corresponding to the expected size was obtained in the WT strain, but no amplification products were obtained from the interrupted mutants ter1-02, ter1-24, ter1-35, and ter1-40 or from the negative control. (TIF) [file pone.0281251.s004.tif]
