# Supplementary material for: Telomerase RNA plays a major role in the completion of the life cycle in Ustilago maydis and shares conserved domains with other Ustilaginales
Source: PLoS One. 2023 Mar 23;18(3):e0281251. doi: 10.1371/journal.pone.0281251 (PMC10035886; doi:10.1371/journal.pone.0281251)
Supplement: S3 Table — (DOCX) [file pone.0281251.s007.docx]

| **Primer** | **Direction** | **Sequence 5' – 3'** |
| --- | --- | --- |
| Cr8S1-5Upp | Fwd | AAGCGGCCGCTAGAGGGGTGGTTTGGGTTTGGT |
| Cr8S1-5Low | Rev | TCGGATCCGCTGGGTCTCGATGCTTTTCTGTA |
| Cr8S1-3Upp | Fwd | CAGGATCCCTACATCGCGATAGCAGTTTG |
| Cr8S1-3Low | Rev | AAGCGGCCGCGGGTGTGGTCTATTGAACG |
| Template-Upp | Fwd | ACCGCGCCTTACTGTCTGAA |
| Template-Low | Rev | TCCCACGTTCCACTTGCTTC |
| WT-ter1-Upp | Fwd | TCCGCCTTCCCTTCTTTGATTTTG |
| WT-ter1-Low | Rev | GTGGCCGACATGCGCTTTGAAC |
| 5H-Upp | Fwd | GAAGGCGTGTGGCTCGGTTTGTTG |
| 5H-Low | Rev | TAGGAAGGCGGCGGAATCAGTTGG |
| 3H-Upp | Fwd | GGAAACCGACGCCCCAGCACT |
| 3H-Low | Rev | CTCAGCCGGCCTTTATCAGC |
| ter-ALL-5a | Fwd | TGCCTCTACCGCATCTAATA |
| ter-i1A-5a | Fwd | TAGTTGCCTAGTTGCTTCTC |
| ter-i1A-3b | Rev | AACCCAAGTGCAGAGTAAAT |
| ter-i4D-5a | Fwd | ACTGTTTGACAATGAAAGCG |
| ter-i4D-3b | Rev | GGTCTGGATGCTTTTCTGT |
| 5ter-rev1 | Rev | CGCCTGACACGCCTCCAACGAT |
| 5ter-rev2 | Rev | ATCACGCGCCCAAGTCACCCTCTG |
| Tub11 | Fwd | GCTCATCGCGCAGGTGGTCTCG |
| Tub12 | Rev | GGAACTGTCGTGGGCGGCTCATT |

**S3 Table**. Primers used in this study.
