# Supplementary material for: Telomerase RNA plays a major role in the completion of the life cycle in Ustilago maydis and shares conserved domains with other Ustilaginales
Source: PLoS One. 2023 Mar 23;18(3):e0281251. doi: 10.1371/journal.pone.0281251 (PMC10035886; doi:10.1371/journal.pone.0281251)
Supplement: S1 Raw images — (PDF) [file pone.0281251.s008.pdf]

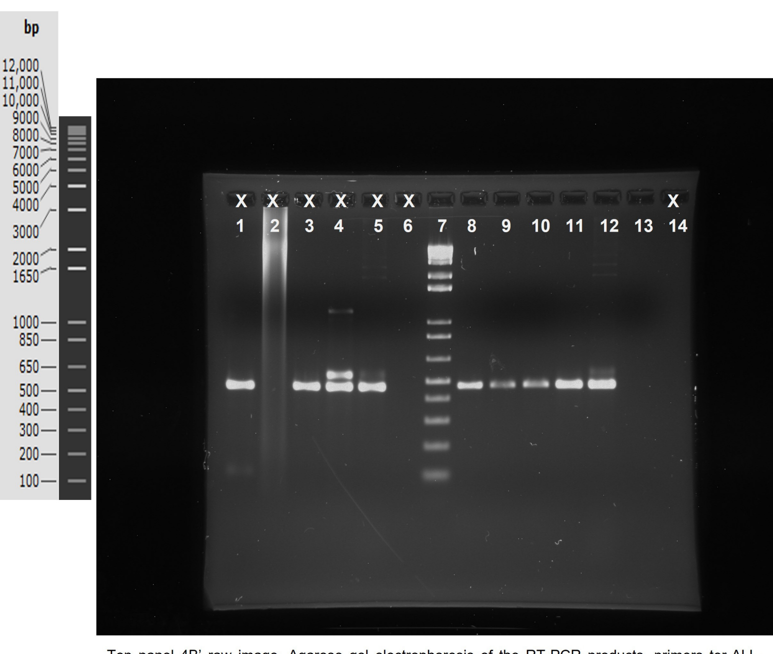

Top panel 4B’ raw image. Agarose gel electrophoresis of the RT-PCR products, primers ter-ALL-5a/Template-Low. Lane 7: 1 kb Plus DNA Ladder (Invitrogen, Carlsbad, CA, USA), samples: cDNA dilution 1:10 and 1:100, obtained with reverse primer WT-ter1-Low (lanes 1 and 8, respectively); cDNA dilution 1:10 and 1:100, obtained with Oligo dT (lanes 2 and 9, respectively); cDNA dilution 1:10 and 1:100, obtained with Random Primers (lanes 3 and 10, respectively); cDNA dilution 1:10 and 1:100, obtained with reverse primer 5ter-rev1 (lanes 4 and 11, respectively); C+ from genomic DNA (lanes 5 and 12); negative control (lanes 6 and 13); empty lane (lane 14). Lanes 8-13 were selected to generate the top panel Figure 4B’. The photography was captured with an imaging system UVP Bio-Doc-it® 220.

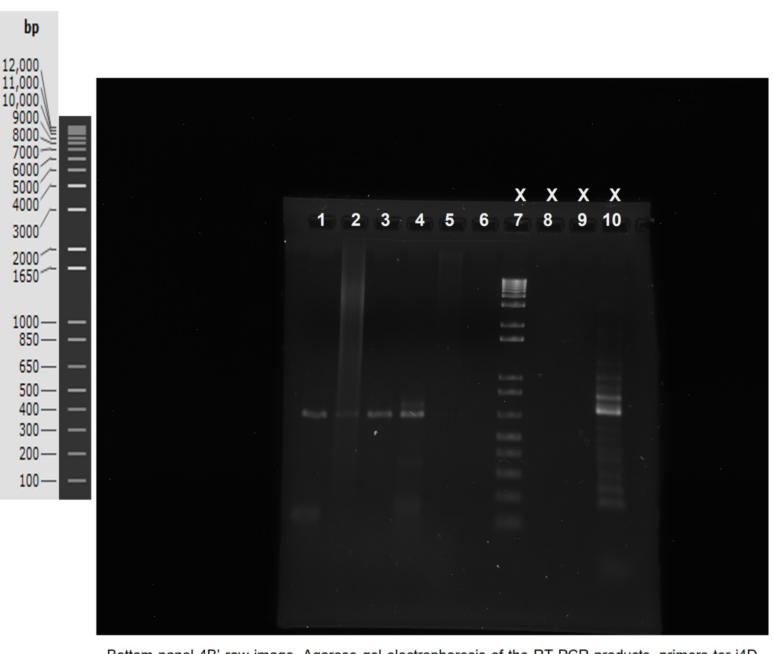

Bottom panel 4B’ raw image. Agarose gel electrophoresis of the RT-PCR products, primers ter-i4D-5a/Template-Low. Lane 7: 1 kb Plus DNA Ladder (Invitrogen, Carlsbad, CA, USA), samples: cDNA obtained with reverse primer WT-ter1-Low (lane 1); cDNA obtained with Oligo dT (lane 2); cDNA obtained with Random Primers (lane 3); cDNA obtained with reverse primer 5ter-rev1 (lane); C+ from genomic DNA (lane 5); negative control (lane 6); empty lane (lanes 8 and 9); sample unrelated to this work (lane 10). Lanes 1-6 were selected to generate the bottom panel Figure 4B’. The photography was captured with an imaging system UVP Bio-Doc-it® 220.

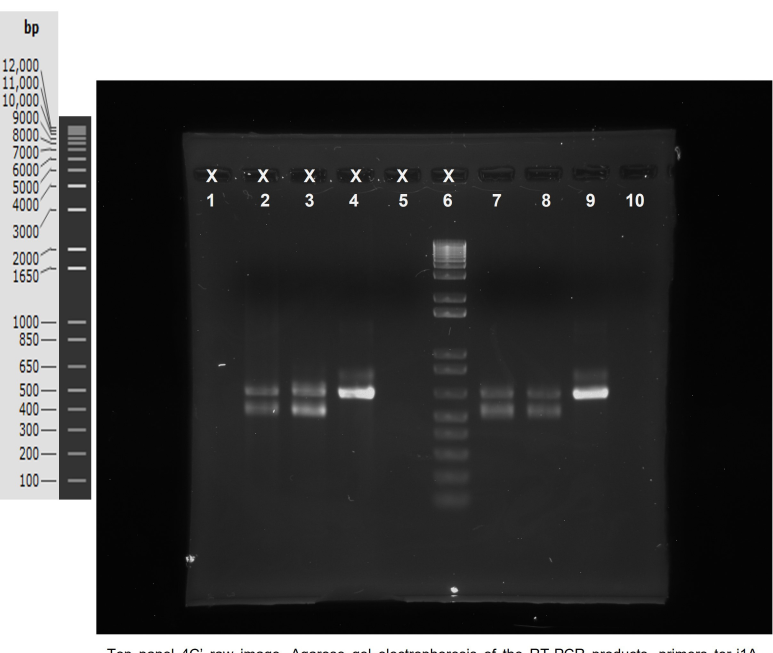

Top panel 4C’ raw image. Agarose gel electrophoresis of the RT-PCR products, primers ter-i1A-5a/5ter-rev2. Lane 6: 1 kb Plus DNA Ladder (Invitrogen, Carlsbad, CA, USA), samples: cDNA dilution 1:10 and 1:100, obtained with Oligo dT (lanes 2 and 7, respectively); cDNA dilution 1:10 and 1:100, obtained with reverse primer 5ter-rev1 (lanes 3 and 8, respectively); C+ from genomic DNA (lanes 4 and 9); negative control (lanes 5 and 10). Lanes 7-10 were selected to generate the top panel Figure 4C’. The photography was captured with an imaging system UVP Bio-Doc-it® 220.

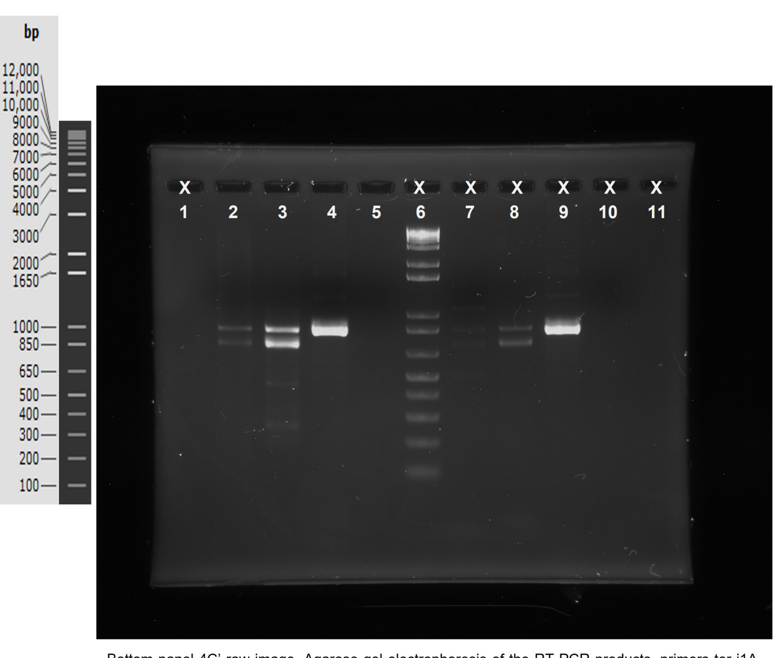

Bottom panel 4C’ raw image. Agarose gel electrophoresis of the RT-PCR products, primers ter-i1A-5a/5ter-rev1. Lane 6: 1 kb Plus DNA Ladder (Invitrogen, Carlsbad, CA, USA), samples: empty lane (lanes 1 and 11); cDNA dilution 1:10 and 1:100, obtained with Oligo dT (lanes 2 and 7, respectively); cDNA dilution 1:10 and 1:100, obtained with reverse primer 5ter-rev1 (lanes 3 and 8, respectively); C+ from genomic DNA (lanes 4 and 9); negative control (lanes 5 and 10). Lanes 2-5 were selected to generate the bottom panel Figure 4C’. The photography was captured with an imaging system UVP Bio-Doc-it® 220.

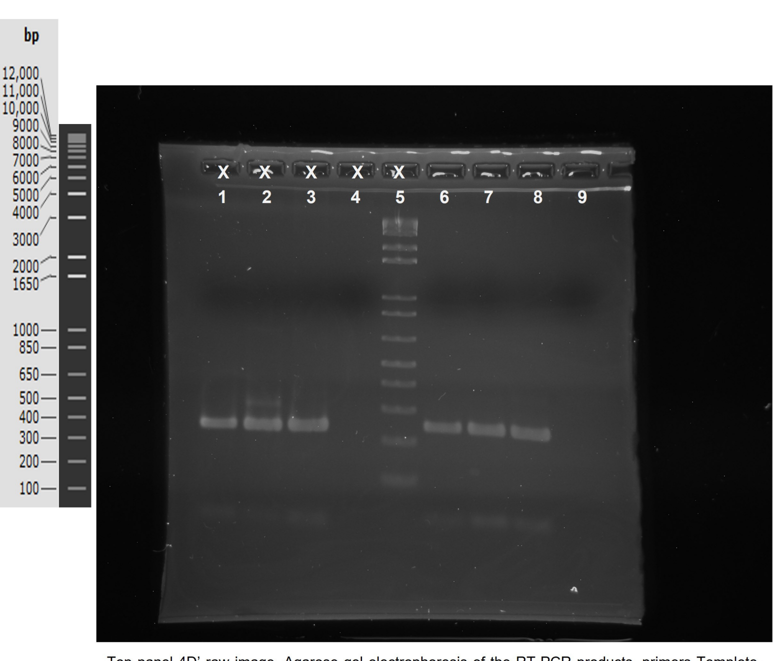

Top panel 4D’ raw image. Agarose gel electrophoresis of the RT-PCR products, primers Templete-Upp/Templete-Low. Lane 5: 1 kb Plus DNA Ladder (Invitrogen, Carlsbad, CA, USA), samples: cDNA dilution 1:10 and 1:100, obtained with Oligo dT (lanes 1 and 6, respectively); cDNA dilution 1:10 and 1:100, obtained with reverse primer 5ter-rev1 (lanes 2 and 7, respectively); C+ from genomic DNA (lanes 3 and 8); negative control (lanes 4 and 9). Lanes 6-9 were selected to generate the top panel Figure 4D’. The photography was captured with an imaging system UVP Bio-Doc-it® 220.

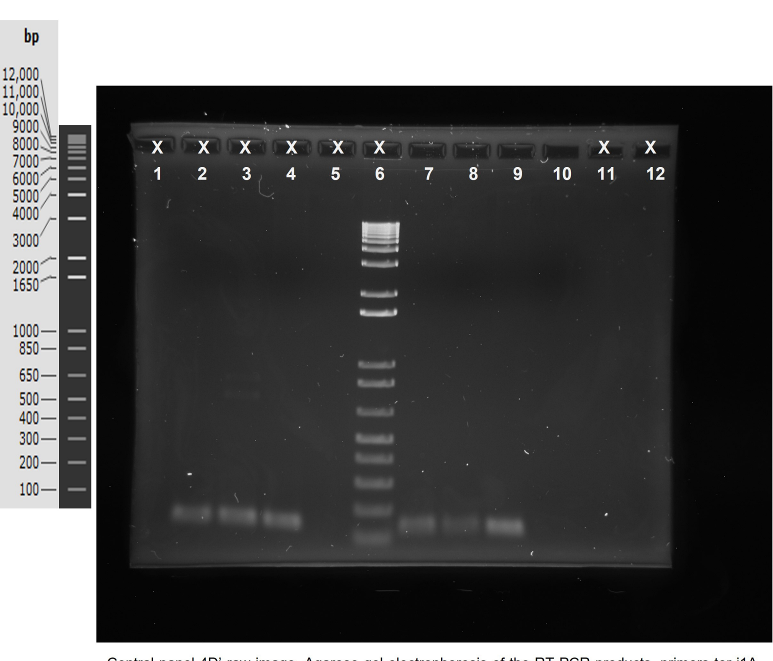

Central panel 4D’ raw image. Agarose gel electrophoresis of the RT-PCR products, primers ter-i1A-5a/ter-i1A-3b. Lane 6: 1 kb Plus DNA Ladder (Invitrogen, Carlsbad, CA, USA), samples: cDNA dilution 1:10 and 1:100, obtained with Oligo dT (lanes 2 and 7, respectively); cDNA dilution 1:10 and 1:100, obtained with reverse primer 5ter-rev1 (lanes 3 and 8, respectively); C+ from genomic DNA (lanes 4 and 9); negative control (lanes 5 and 10); empty lane (lanes 11 and 12). Lanes 7-10 were selected to generate the central panel Figure 4D’. The photography was captured with an imaging system UVP Bio-Doc-it® 220.

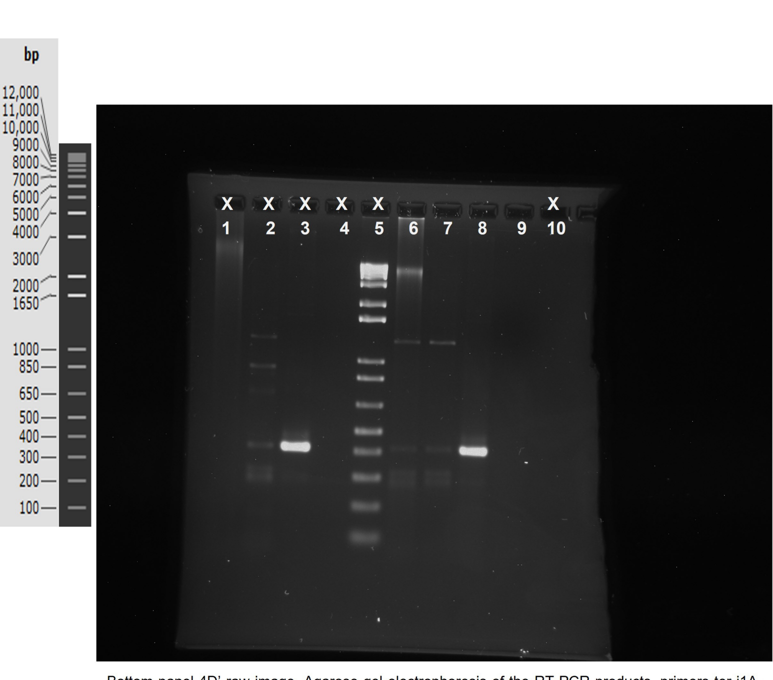

Bottom panel 4D’ raw image. Agarose gel electrophoresis of the RT-PCR products, primers ter-i1A-5a/ter-i4D-3b. Lane 5: 1 kb Plus DNA Ladder (Invitrogen, Carlsbad, CA, USA), samples: cDNA dilution 1:10 and 1:100, obtained with Oligo dT (lanes 1 and 6, respectively); cDNA dilution 1:10 and 1:100, obtained with reverse primer 5ter-rev1 (lanes 2 and 7, respectively); C+ from genomic DNA (lanes 3 and 8); negative control (lanes 4 and 9); empty lane (lane 10). Lanes 6-9 were selected to generate the bottom panel Figure 4D’. The photography was captured with an imaging system UVP Bio-Doc-it® 220.

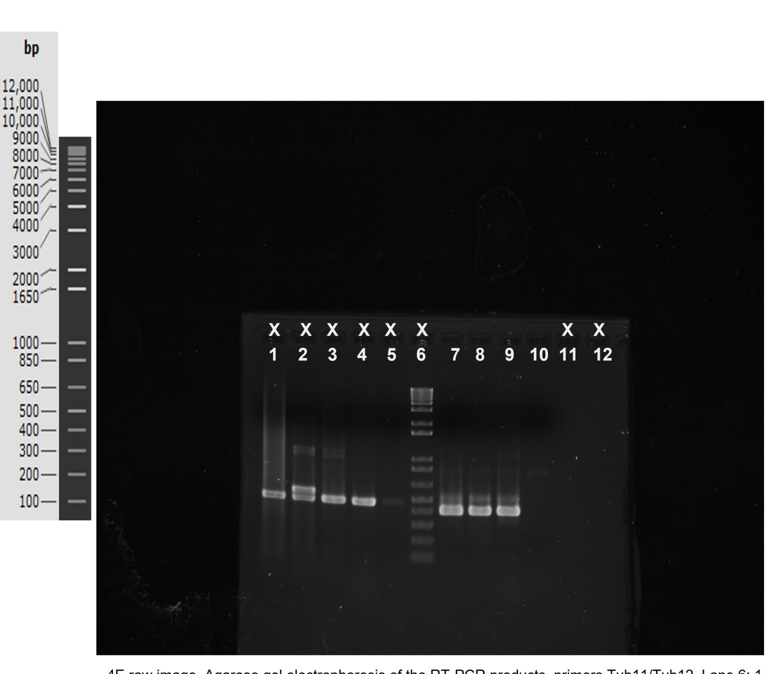

4E raw image. Agarose gel electrophoresis of the RT-PCR products, primers Tub11/Tub12. Lane 6: 1 kb Plus DNA Ladder (Invitrogen, Carlsbad, CA, USA), samples: cDNA obtained with Oligo dT (lane 7); cDNA obtained with reverse primer Tub12 (lane 8); C+ from genomic DNA (lane 9); negative control (lane 10): empty lane (lanes 11 and 12); samples unrelated to this work (lanes 1-5). Lanes 7-10 were selected to generate the Figure 4E. The photography was captured with an imaging system UVP Bio-Doc-it® 220.

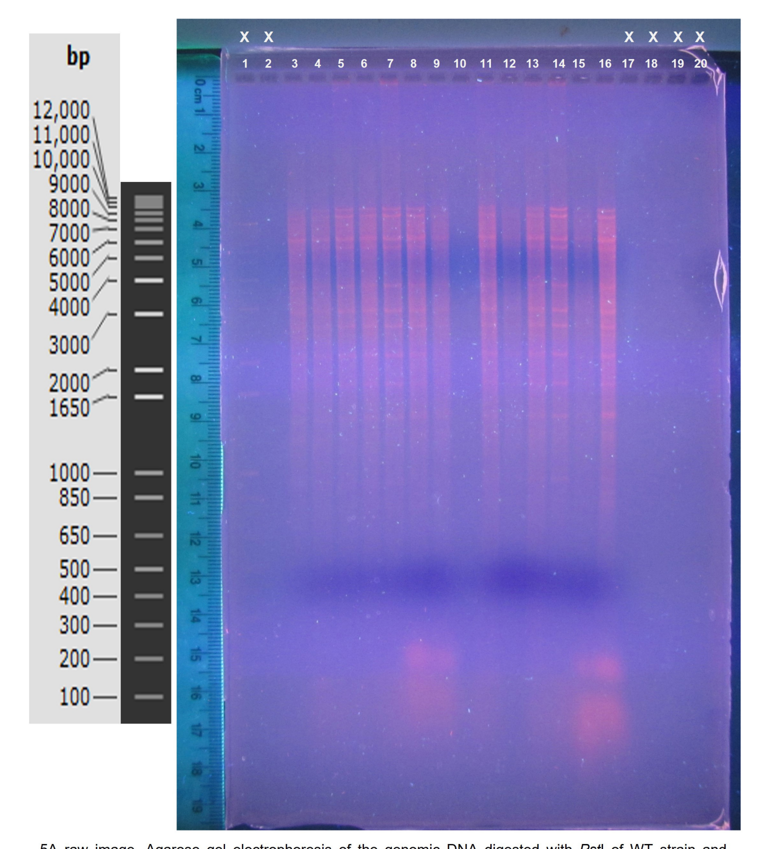

5A raw image. Agarose gel electrophoresis of the genomic DNA digested with *Pst*I of WT strain and interrupted transformants. Lane 1: 1 kb Plus DNA Ladder (Invitrogen, Carlsbad, CA, USA), samples: WT strain (lane 3); ter1-02 (lanes 4-9); ter1-24 (lanes 11-16); empty lanes (lanes 17-20). Lanes 3-16 were selected to generate the Figure 5A. The photography was captured with Canon PowerShop SX210 IS camera.

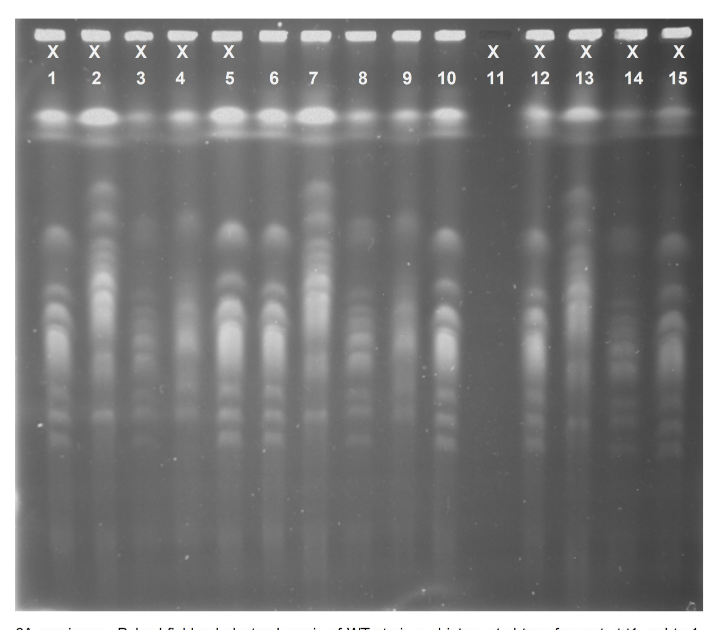

6A raw image. Pulsed-field gel electrophoresis of WT strain and interrupted transformants trt1 and ter1. Samples: WT strain (lanes 1, 6 and 12); trt1-1 (lanes 2, 7 and 13); trt1-2 (lanes 3, 8 and 14); ter1-02 (lanes 4 and 9); ter1-24 (lanes 5, 10 and 15). Lanes 6-10 were selected to generate the Figure 6A. The photography was captured with an imaging system UVP Bio-Doc-it® 220.

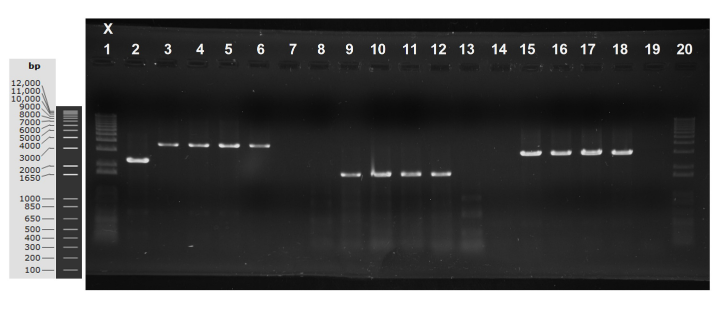

S4-C raw image. Agarose gel electrophoresis of PCR amplified products from the genomic DNA of WT strain and interrupted transformants. Lane: 1 and 20: 1 kb Plus DNA Ladder (Invitrogen, Carlsbad, CA, USA), samples: WT strain (lanes 2, 8, 14); ter1-02 (lanes 3, 9, 15); ter1-24 (lanes 4, 10, 16); ter1-35 (lanes 5, 11, 17); ter1-40 (lanes 6, 12, 18); Control (-) lanes (7, 13, 19). Lanes 2-20 were selected to generate the Figure S4-C. The photography was captured with an imaging system UVP Bio-Doc-it® 220.

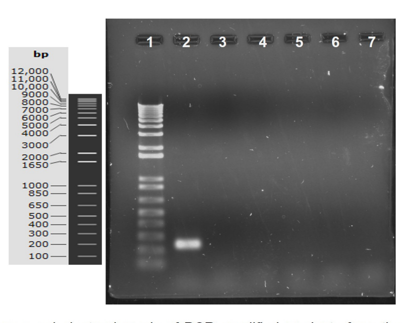

S4-D raw image. Agarose gel electrophoresis of PCR amplified products from the genomic DNA of WT strain and interrupted transformants. Lane 1: 1 kb Plus DNA Ladder (Invitrogen, Carlsbad, CA, USA), sample: WT strain (lane 2); ter1-02 (lane 3); ter1-24 (lane 4); ter1-35 (lane 5); ter1-40 (lane 6); Control (-) lane (7). Lanes 1-7 were selected to generate the Figure S4-D. The photography was captured with an imaging system UVP Bio-Doc-it® 220.
