# Supplementary material for: Telomerase RNA plays a major role in the completion of the life cycle in Ustilago maydis and shares conserved domains with other Ustilaginales
Source: PLoS One. 2023 Mar 23;18(3):e0281251. doi: 10.1371/journal.pone.0281251 (PMC10035886; doi:10.1371/journal.pone.0281251)
Supplement: Attachment — Submitted filename: To editor PLoS-18-0ct-2022.docx [file pone.0281251.s009.docx]

**Editor's comments:**

Thanks to the reviewers for the kind review of the manuscript "Telomerase RNA plays a major role in the completion of the life cycle in *Ustilago maydis* and shares conserved domains with other Ustilaginales*”* by Sanpedro-Luna *et al.*

The authors, respectfully, would like to address the reviewers' concerns and proposed changes.

**Northern and qPCR studies must be conducted to provide data for the presence of isoforms.**

Dear Sir, instead of qPCR studies, we conducted RT-PCR assays using a series of primer pairs to demonstrate the presence of the predicted isoform; the existence of the fragments originated from the transcript isoform is shown in Fig 4 and stated in lines 536–586 of the manuscript.

**Furthermore, qPCR studies must be done to verify the presence of polycistronic messages. This has not been observed in this genus before and the evidence cannot be based on RNA-seq data alone.**

The occurrence of natural polycistronic transcripts was corroborated alongside the finding of the RT-PCR products in the expected sizes when oligo(dT) and specific reverse oligonucleotide-primers were used to synthesize the first cDNA strand; the PCR primer-pairs used to amplify the transcript isoforms, those spanned from UMAG_03168 to *ter1* regions. The findings included transcripts in the size of continuous exon and intron sequences as is in the Genomic sequences and fragments in the sizes of spliced isoform sequences. Multiple banding shown in Fig.4 corresponds to the isoforms processed by alternative splicing; these results are detailed in lines 536–586 of the manuscript.

**Differences in the kinetics of survival vary from experiment to experiment and it is inappropriate to compare survival kinetics between the authors' and other laboratories. The experiments must be carried out side by side**

The conclusions of the differences between the kinetics of the *trt1Δ* mutants and *ter1* have been corrected.

**A set of second issues deals mostly with presentation issues.**

**UmTay1 IS NOT the U. maydis version of human Trf. This suggestion is incorrect.**

The statement referring to UmTay as the homolog of Trf1 has been removed, and correction is stated in lines 112–121, which further justifies the work done and the importance of *ter1* and *trt1.*

**The structure of the Sm binding region of the RNA is atypical. The authors must discuss this finding.**

The atypical structure of the Ustilaginales Sm domain is discussed in lines 747–754 according to the results of Jones and Guthrie, 1990 and Kuprys et al., 2013.

**You must present bioinformatic studies with appropriate qualifications or exclude them from this paper. Regardless, the experimental data must stand on its own.**

Bioinformatic studies provide primary evidence of *ter1-*containing alternative isoforms. Thus we resubmit the bioinformatic analysis to editor consideration with additional information: studies were conducted with the Trinity Pipeline Grabherr et al. 2011, with the parameters suggested by the authors and those previously reported by Donaldson et al. 2017, as detailed in the materials and methods section lines 245–269, raw sequencing reads and expression are available from the Gene Expression Omnibus of NCBI (accession number: GSE184051).

**Present the paper in a hypothesis-driven fashion and separate each section of the paper to provide greater fluency which is necessary for proper communication of the data. The Reviewers suggest a number of specific suggestions in this regard.**

**Please ensure that you follow the PLOS One guidelines for manuscript preparation.**

**Please address all of the remaining issues from each reviewer.**

**I concur with the viewpoints of the Reviewers.**

**Specific Comments:**

**Reviewer #1:**

**1. The authors named the RNA gene *ter114*, presumably because this is their candidate #114. However, it is better to rename the gene ter1 to be consistent with standard convention and to eliminate confusion.**

The gene has been renamed *ter1* to match standard gene nomenclature.

**2. Introduction, line 99. It is not advisable to refer to UmTay1 as the U. maydis version of human Trf1. The proteins have very different domain structures and are probably not evolutionarily related. Even though both UmTay1 and human TRF1 bind to and regulate Blm helicase, it is misleading to suggest that they are equivalent.**

The statement referring to UmTay as the homolog of Trf1 has been removed and replaced by lines 112–121, which further justifies the work done and the importance of *ter1* and *trt1.*

**3. The unusual mode of synthesis for *U. maydis* telomerase RNA and the existence of alternative isoforms require further analysis. If I understand correctly, the conclusions regarding the transcripts in the relevant region of the genome are based entirely on RNA-seq analysis. Since no other fungal telomerase RNA is known to be synthesized via read-through transcription of another gene, it is important to substantiate this idea with an alternative approach. For example, the authors can reverse transcribe the RNA preparation using a primer near the ter1 template, and then use primers flanking the introns of UMAG_03168 to amplify predicted isoforms of the composite transcripts. Alternatively, they could use S1 or RNase protection mapping to confirm the existence of these transcripts.**

The suggestion has been heeded; the results of the RT-PCR assays are detailed in lines 536–586 of the manuscript and in Fig.4.

**4. Line 592-596. The authors compared the kinetics of ter1 survivor formation in the current study to that for trt1 in a previous paper, and concluded that ter1 survivors emerged earlier. However, there is significant variability in the process of survivor formation; a side-by-side comparison using multiple, independent population of the mutants will be needed to support this claim. Alternatively, the authors can soften their conclusion.**

The conclusions of the differences between the kinetics of the *trt1* mutants and *ter1* mutants have been corrected.

**5. There are numerous instances of awkward English throughout the manuscript. The authors are encouraged to obtain more editorial assistance.**

The revised version of the manuscript contains suggested changes.

**Reviewer #2**

**In this manuscript, Sanpedro Luna et al. report that they have found the telomerase RNA subunit gene in the fungus Ustilago maydis. The authors do experiments in this species to test if their search for the telomerase RNA gene in the broader set of Ustilaginaceae family has identified this core telomerase RNP subunit. The experiments include mutating the gene partially and checking telomere length in this organism as well as other phenotypes. There were some potentially interesting findings in this regard, both individually and compared to mutations in the TERT homolog in *U. maydis*. From what I can tell, I think that it is probable that the authors have found the telomerase RNA gene in this organism (and probably all of the listed related species), based on the alignments along with the telomere-length shortening and other growth phenotypes of the truncation mutant allele. However, it is also not convincing as to what the bounds of the transcript(s) are, the abundance of these RNA species, how the proposed template might lay down *U. maydis* telomeric DNA repeats (are these repeats known?), etc.**

Previously, Guzmán and Sánchez (1994) published the sequences of the terminal restriction fragment (TRF) of *U. maydis*. There they determined that the telomeric repeated motif TTAGGG of *U. maydis* is shared with that of vertebrates; based on this premise, the mining of sequences within the genome of the fungus was carried out using the CCCTAA motif to identify all the regions containing it and to select the potential candidates according to the synteny analysis.

**The authors provide no experimental data of their own (that is actually shown) in this manuscript confirming ter114 RNA isoform(s) are detectable/exist. This is such a major weak point, along with the cursory analysis of the putative template and how conserved patches might relate to some overall secondary structure, that it is hard to appreciate validity of later figures.**

RT-PCR assays were conducted to verify the presence of polycistronic transcripts without interruptions or processed by alternative splicing. Those transcripts contain UMAG_03168 sequences at their 5’-end, and the *ter1* encoding region harboring the template motif at the 3', 536 to 586 rows of the manuscript. The poor conservation of the nucleotide sequences and variations in the length of the intergenic regions, together with the lack of proof of existence of polycistronic transcript, or the transcription start sites in the orthologous loci of the other Ustilaginales species made, at these times, impossible to estimate a *ter1* secondary structure for the entire taxon, limiting our study to the prediction of folding sequences that were well conserved such as pseudoknot domain and P6.1 loop.

**These weaknesses could be addressed with showing RT-PCR results and, if at all possible, a successful northern blot. (As for some of the nuances about mutant phenotypes beyond the telomere Southern blot, I am not sure that I can really critique them, since I am not an Ustilagginaceae expert.)**

Suggested RT-PCR assays were performed and are detailed in lines 536–586 of the manuscript and in Fig.4.

**There are weaknesses also with the putative gene’s structure; e.g., why is there a C instead of a G in the proposed Sm-binding consensus sequence?**

The comments about the Ustilaginales Sm domain's atypical structure are discussed in lines 747–754 according to the results observed by Jones and Guthrie, 1990 and Kuprys et al., 2013.

**And can the authors propose any sort of secondary structure that might help explain the observed conserved patches in the alignment? They have clearly been using RNAalifold and presumable secondary-structure predictions, so how much covariation supports possible models? The 5’ and 3’ boundaries of the transcript(s) are important for this, however, and these are not clear from data presented, so the authors should figure this out to inform bioinformatic folding modeling. From what is provided, I cannot assess the validity of the authors claims. Then, from Figures 4 through 6, there are additional points of interest, but it leads to a broad but only sporadically well-supported set of data underlying the authors overall story.**

The poor conservation of the nucleotide sequences and variations in the length of the intergenic regions, together with the absence of evidence of polycistronic transcripts or transcription start sites in close species, impede to estimate *in silico* a general secondary structure for all the Ustilaginales here included. The estimation of the folding of the pseudoknot domain was performed manually considering the criteria described by Gunisova et al., 2009, for the folding of pseudoknots. Dr. Julian Chen, in personal communication, let us know the length of 1291 nt. of the transcript, which is in the NCBI GenBank in March with an accession number ON015423 (https://www.ncbi.nlm.nih.gov/nuccore/ON015423), and currently is waiting for publication.

Logeswaran, D., Y. Li, K. Akhter, J.D. Podlevsky, T.L. Olson, K. Forsberg and J.J.-L. Chen (2022) Biogenesis of telomerase RNA from a protein-coding mRNA precursor. Proc. Natl. Acad. Sci. U.S.A. (in press)

**The Results section is written at times like a Methods section, and at other times like an Introduction section. It is too long and rambling and it could also use subheaders to guide the reader easily through what the main biological findings are supporting the claims by the authors. As written, it is very hard to read from end to end with some sort of a sense of focus and a story. Part of the issue is that it vacillates between focusing on important results and then overly extensive methods details. The Results section could use more structure that sticks closer to the scientific method: what are the authors’ hypotheses and conclusions as we read through it? The rationale should be more explicitly stated, both early and often, in each section. As for the part that reads as an Introduction, this is lines 92–112.**

The review and restructuring of the manuscript content were performed.

**Also, the figure legends being hard to differentiate from the Results text is another issue. What would be helpful is to put the legends also under the figures, in addition to where they are requested to be by the journal. If they are to stay in the Results, it would help to indent these sections at least, or find some way to make them stand out. How does PLoS One instructions to authors want this formatted?**

Paragraph indentation has been adjusted, and figure legends have been placed immediately at the end of each paragraph where reference is made to comply with submission guidelines.

**My understanding of Sm-binding sites is that there is an important guanosine after the stretch of U’s (e.g., see Seto et al., Nature 1999). Why does the proposed Sm-binding consensus in these fungal species (AAU(_5-6_)CY) have a C at this position instead of G? This seems surprising and concerning; the authors should do more to explain, ideally with additional data, why this is a bona fide Sm7-binding site and not just some other conserved patch that coincidentally approximates an Sm consensus site. Line 469: how is it that “Fig 2A and 2B” indicated show any evidence of the putative Sm site having a functional importance based on schematics and alignments? The Results here overstate what data the authors have/show; the text seems to refer to other papers’ data on other species telomerase RNAs, not this manuscript’s experimental results, which are too limited to support all of the authors’ claims, even though the alignments generally look tantalizing for the most part to this reviewer.**

The atypical structure of the Ustilaginales Sm domain is discussed in lines 747–754 according to the results observed by Jones and Guthrie, 1990 and Kuprys et al., 2013.

**Lines 489–542 are far too speculative about ter114 expression from transcriptomic datasets. The authors choose to connect the reported forms of RNAs from the locus with splicing forms and it is mixed with protein-coding mRNAs, and I find it confusing, overly long, and weak.**

Evidence for polycistronic transcripts obtained by RT-PCR assays is detailed on lines 536–586 of the manuscript.

**Figure 3 is unscientific: there are cartoons showing what the authors try to convey as ter114 transcripts, but I can’t understand it; what is the scale of B compared to A in Figure 3? Why can’t the authors show us a northern blot of the ter114 transcripts that seem suggested by RNA-seq data in A? What does ter114 have to do with the flanking mRNAs? It is all tantalizing, but too confusing and unconvincing due to very limited new experimental data provided by these authors. I think the authors should shorten and lessen the focus on the bioinformatic approach mining RNA-seq datasets, clarify and simplify impactful main points, and focus more on experimental testing of the transcription instead; i.e., building up what was started by RT-PCR in lines 543–551. In this section, the authors must show their RT-PCR results and address this issue:**

Suggestions have been addressed; however, we chose to do PCR assays.

**Lines 547, 550: “(data not shown)”… does PLoS One allow data not shown?**

**This section on the RT-PCR tests for ter114 transcript(s) also needs simplifying; readers don't need to know names of primers used, etc. It is too cumbersome to have to read all this peripheral detail; any major points get lost in the methodological peripheral details. Again, have the authors tried to simply do a northern blot on total RNA to detect the ter114 isoform(s)? This would help the authors’ entire manuscript tremendously. This is not just because it is a canonical method, but because it would show so much more information, quantitatively, about numbers of isoforms, they could see how the truncation allele they generated affects them, etc. This is hard to do by the more hunt-and-peck approach of RT-PCR amplicons, and more convincing than the jumpy RNA-seq tracks, perhaps due to low coverage of the transcriptome.**

Suggestions have been addressed.

**Lastly, the authors searched for telomerase RNA using 1.5 copies of CCCTAA. Is the actual telomeric repeat in this organism known? What inspired the authors to exactly choose this sequence? The authors have the putative template sequence in ter114, but do not explain how exactly this sequence could lay down the telomeric DNA repeats in this organism, or if the telomere repeats are unknown exactly. They should propose some sort of catalytic model that gives some confidence that the template found could indeed dictate the telomere repeat sequences in this organism.**

Previously Guzmán and Sánchez (1994), by sequencing libraries of the terminal restriction fragment of chromosomes, determined that the telomeric repeat of *U. maydis* is identical to that of vertebrates containing the TTAGGG motif; with this consideration, the mining of sequences was carried out using the CCCTAA motif within the genome of the fungus to identify all the regions. We used 1.5 stretches long after analyzing template sequences in the TelomeraseDB page (<http://telomerase.asu.edu/>) to select potential candidates based on the synteny analysis.

**Overall, this is a potentially exciting story about the identification of the telomerase RNA gene in Ustilaginaceae, yet it is presented as a data-diffuse and insufficiently rigorous (and overly long and technical) manuscript format that make it hard to feel confident in the authors tantalizing claims. Hopefully the authors can add more convincing data about ter114 transcriptional isoform(s) by adding a northern blot and/or showing a lot of RT-PCR data and make the manuscript’s Results section and figures more cogent. This would greatly improve this manuscript.**

**Minor:**

**Subject-verb agreement problem in the title: should be “shares”.**

Suggestions have been addressed.

**Line 454: I do not understand why the authors think that CE1 and CE2 are likely to be promoter elements rather than structural elements in the encoded TER114 RNA.**

At the beginning of the work, the lack of knowledge that *ter1* originates from a polycistronic RNA led us to search the promoter regions of the gene within the intergenic region. We found CE1 element upstream of the template domain, which resembles the binding site for ZF proteins according to MatInspector software (Genomatix, <https://www.genomatix.de>), and its conservation within the taxonomic group, led us to speculate that it could be the promoter of *ter1*. This idea was then discarded and now supported the idea by RT- PCR that they are only a part of the transcript.

**Line 462: the P6.1-like element is not essential in *S. cerevisiae* (Zappulla et al., NSMB 2005). The authors should not overstate its necessity.**

Suggestions have been addressed.

**Line 491: “the the”**

The sentence has been corrected.

**Overall, the writing is good, except in the Acknowledgements, where editing is necessary.**

Suggestions have been addressed.

Thakn you so much for your time and consideration,

Sincerely

Patricia Sánchez
