# Supplementary material for: Telomerase RNA plays a major role in the completion of the life cycle in Ustilago maydis and shares conserved domains with other Ustilaginales
Source: PLoS One. 2023 Mar 23;18(3):e0281251. doi: 10.1371/journal.pone.0281251 (PMC10035886; doi:10.1371/journal.pone.0281251)
Supplement: Attachment — Submitted filename: Responses to Reviewers.docx [file pone.0281251.s010.docx]

To the Editor:

Thank you and to the reviewer for the time and dedication spent in the review of this paper; we acknowledge all the work.

We have accomplished virtually all the requirements from Prof. Dr. Barry Saville.

In relation to *ter1Δ* complementation, it is technically unfeasible to do so because of the great lability of this kind of survivor; in our hands, the frailty of *ter1Δ* survivors is deeper than that of their *trt1Δ counterparts.* The additional challenges to do so relied in the deregulation of genes involved in the cell wall composition (among others) that practically impede the obtention of living protoplasts to achieve gene transformation; mutant cells also die after prolonged times of incubation in cell wall lysing enzymes (which are toxic). Protoplast/DNA fusion with PEG also requires temperature changes that *ter1Δ* mutants are feeble to endure, and finally, the replication times are large. We could assume 8 hours of incubation in rich medium to start the chimeric *ter1* expression in transformants; this is true in wild-type cells, but long days would be required to obtain one or no transformants after several experiments.

However, we follow the work of Logeswaran et al., 2022. Proc. Natl. Acad. Sci. USA to reinforce our finding of the telomerase RNA subunit of *Ustilago maydis.*

Regarding the other minor points the authors may consider addressing, which are wording issues and matters of sentence clarity, and you gently have highlighted, they have been reviewed.

**Lines 117-121 Clarify, when does the repression of the putative TER gene occur perhaps break the sentence into two to help clarify the points being presented**

It has been edited; thank you for the suggestion.

**Line 126 the phrase "order representatives here studied" is awkward and not clear, edit for clarification**

It has been edited; thank you for the advice.

**Lines 237-239 After running the sucrose cuchion centrifugation were samples assessed to determine the purity and degree of subcellular fractionation, i.e., how pure were the nuclei isolated in this manner?**

Nuclei were harvested in a small fraction as described in the text and treated as described by Groner and Phillips (1975). The nuclei were recognized in a small sample stained with propidium iodide under a fluorescence microscope. Staining was performed as described in Alfa et al., 1993. *In*: Experiments with fission yeast. A laboratory course manual. Experiments 1 and 2. CSHL Press NY USA. Nuclei were harvested with mitochondria; however, after total RNA extraction and ribodepletion of the nuclei fraction, the sequenced transcriptome was useful for this work, as it had the read number required to publish after filtering for mitochondrial transcripts. Because no quantitative comparisons were made against total cytoplasmic or total cellular RNA, no highly purified nuclei were required in this work.

**Lines 550-554 The portion of the sentence beginning "ratifying..." is not clear and could be edited to improve clarity. Additionally, consider breaking up this overly long sentence**

It has been edited; thank you for the suggestion.

**Lines 581-582 "results points to the fact" should be edited to results suggest or results support an RNA maturtion process that involves.**

It has been edited; thank you for the suggestion.

**Lines 583-586 The portion of the sentence " ...so unlike TLC1... " seems like discussion not results. Consider moving this to the discussion.**

The text fragment has been moved to lines 766-770; thank you for the suggestion.

**Line 612 the phrase "every at approximately 24 duplication rounds, " is not clear edit to clarify what you intend to convey**

It has been edited to clarify; thank you for the suggestion.

**Line 649 and elsewhere the phenotypic differences that distinct mutants present lead me to ask - Were there any DNA-level differences detected between the mutants that might help explain mutant to mutant variation?**

There is a point mutation in the same locus that is under study; perhaps is speculation, however, we require complete genome sequencing of the strains to discard any other external effects. As stated by Chen et al. and us, those loci are rich in (apparently functional) transcript isoforms.

**Line 687 has should be had**

It has been edited; thank you for the advice.

**Line 751 should " extended a pair" be extended by a pair?**

It has been corrected; thank you for the advice.

**Lines 752-754 wording of this sentence could be improved eg denotate does not seem to be a good word choice here.**

The text has been edited; thank you so much.

**Lines 757-759 the sentence beginning "Whether ..." is not clear and could be edited to improve clarity**

Your suggestion has been addressed. Thank you

**Lines 796-798 This sentence is not clear edit to better explain what you are trying to convey**

It has been edited; thank you for the suggestion.

We hope that the manuscript's edition will make it suitable for publication in your prestigious journal; we appreciate your time and dedication to this work.

Thank you sincerely,

Dr. Patricia Sánchez Alonso

Corresponding Author
